# Thermal Investigations of Annelated Triazinones—Potential Analgesic and Anticancer Agents

**DOI:** 10.3390/molecules28186542

**Published:** 2023-09-09

**Authors:** Małgorzata Sztanke, Krzysztof Sztanke, Agnieszka Ostasz, Halina Głuchowska, Renata Łyszczek

**Affiliations:** 1 Department of Medical Chemistry, Medical University of Lublin, 4A Chodźki Street, 20-093 Lublin, Poland; malgorzata.sztanke@umlub.pl; 2 Laboratory of Bioorganic Compounds Synthesis and Analysis, Medical University of Lublin, 4A Chodźki Street, 20-093 Lublin, Poland; 3 Department of General and Coordination Chemistry and Crystallography, Faculty of Chemistry, Institute of Chemical Sciences, Maria Curie-Skłodowska University, M.C. Skłodowskiej Sq. 2, 20-031 Lublin, Poland; agnieszka.ostasz@mail.umcs.pl (A.O.); halina.gluchowska@mail.umcs.pl (H.G.); renata.lyszczek@mail.umcs.pl (R.Ł.)

**Keywords:** disubstituted annelated triazinones, analgesic agents, anticancer agents, thermal characterisation, thermal behaviour, thermal degradation mode, TG-DSC, TG-FTIR, toxicity to erythrocytes, antihaemolytic activity

## Abstract

In this article, for the first time, TG-DSC and TG-FTIR investigations of potential pharmaceutics, i.e., analgesic and anticancer active annelated triazinones (**1**–**9**) have been presented. The thermal behaviour of these molecules was established in oxidative and inert conditions. The solid–liquid phase transition for each compound (**1–9**) was documented by one sharp DSC peak confirming the high purity of each sample studied. All the molecules were characterised in terms of calorimetric changes and mass changes during their heating. They revealed high thermal stability in oxidative and inert conditions. The observed tendency in thermal stability changes in relation to a substituent present at the phenyl moiety was found to be similar in air and nitrogen. It was confirmed that annelated triazinones **1–9** were stable up to a temperature range of 241–296 °C in air, and their decomposition process proceeded in two stages under oxidative conditions. In addition, it was established that their thermal stability in air decreased in the following order of R at the phenyl moiety: 4-Cl > 3,4-Cl_2_ > H > 3-Cl > 4-CH_3_ > 2-CH_3_ > 3-CH_3_ > 2-Cl > 2-OCH_3_. The volatile decomposition products of the investigated molecules were proposed by comparing the FTIR spectra collected during their thermogravimetric analysis in nitrogen with the spectra from the database of reference compounds. None of annelated triazinones **1–9** underwent any polymorphic transformation during thermal studies. All the compounds proved to be safe for erythrocytes. In turn, molecules **3**, **6,** and **9** protected red blood cells from oxidative damage, and therefore may be helpful in the prevention of free radical-mediated diseases.

## 1. Introduction

**Figure 1:**
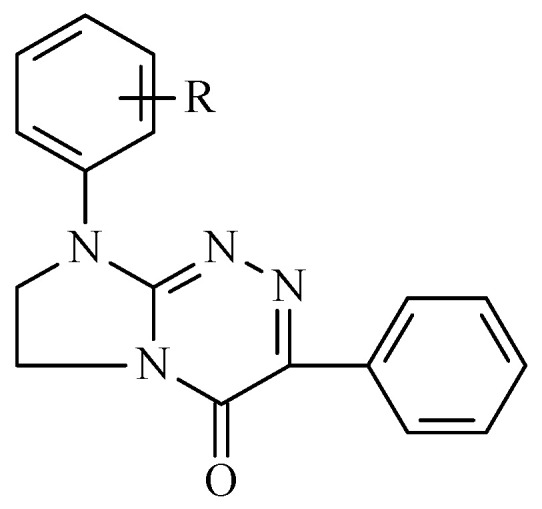
Structures of the studied compounds: **1**. R = H, **2**. R = 2-CH_3_, **3**. R = 3-CH_3_, **4**. R = 4-CH_3_, **5**. R = 2-OCH_3_, **6**. R = 2-Cl, **7**. R = 3-Cl, **8**. R = 4-Cl, **9**. R = 3,4-Cl_2_.

3-Phenyl-8-(R-phenyl)-7,8-dihydroimidazo[2,1-*c*][1,2,4]triazin-4(6*H*)-ones (**1**–**9**) (Figure 1) belong to an important class of disubstituted annelated triazinones, whose structures both in solution and in the solid state have been determined [1,2]. Compounds **3** and **6**–**9** have been patented due to their disclosed analgesic activities in the central nervous system as well as low acute toxicities for mice [1]. Molecules **3** and **7–9** have been shown to be antiproliferative active on human peripheral blood myeloma cells, suggesting their usefulness in chemotherapy of haematological malignancies. Additionally, compounds **3** and **6–8** have been found to exhibit remarkable antimigratory properties in human cervical carcinoma cells, suggesting their antimetastatic potentials [2]. The most selective and therefore the most pharmacologically satisfactory molecule of this class proved to be 3-phenyl-8-(4-chlorophenyl)-7,8-dihydroimidazo[2,1-*c*][1,2,4]triazin-4(6*H*)-one (**8**), which was completely non-toxic in vitro and slightly toxic in vivo [2]. It has been shown in previous studies that all these potential analgesic and anticancer agents are able to easily cross cell membranes, including the blood–brain barrier due to their lipophilicity values which are optimal for favourable pharmacokinetic properties [3,4]. A developed procedure for the quantitative determination of the most selective anticancer annelated triazinone (**8**) has been recently reported. This achievement has been disclosed as the first analytical method with potential applicability in clinical analytics [5].

Knowledge of the thermal behaviour, purity, and the decomposition mechanism of each bioactive compound is already very important in the preliminary and preclinical phase of drug development. Thermal analysis methods are essential not only in studies of molecular pharmaceutics [6,7] but also for drug candidates [8,9] and phase change materials [10,11,12].

Some thermal studies on pharmacologically important fused 1,2,4-triazinones have been previously carried out in our laboratory [13,14]. These scientific investigations were focused mainly on studying their thermolysis and concerned their detailed thermal characterisation. We confirmed that azoloannelated triazines, possessing a completely conjugated six-membered system, were stable at temperatures much higher than ambient conditions. These results justified the rationale for our continuous investigation and thermal characterisation of various classes of diheterocyclic molecules with the conjugated triazinone system; therefore, in light of scientific reports, the pharmaceutical substances stable at temperatures much higher than ambient temperature can be stored at 20–45 °C without fear of losing their shelf life [15,16]. 

However, there is a research gap in the thermal characterisation of annelated triazinones. In particular, no previously conducted thermal studies have concerned molecules such as 3-phenyl-8-(R-phenyl)-7,8-dihydroimidazo[2,1-*c*][1,2,4]triazin-4(6*H*)-ones (**1–9**), which we patented and extensively researched for their possible pharmaceutical application [1,2,3,4]. The thermal properties and the thermal degradation mode of this class of possible analgesics and anticancer agents (**1–9**) are unknown to date, despite the above-mentioned fact that they are still being evaluated as potential drugs and that one of their derivatives (**8**) has a potential use in clinical analytics [5]. Therefore, all these molecules (**1–9**), which are an important class of analytes, were subjected to our thermal studies. 

Hence, the novelty of the present investigation is to determine the thermal features and thermal properties of all these molecules under oxidative and inert conditions. We try to explain the relationships between the structure and thermal properties of the compounds recruited, and to find out how the thermal degradation mode of the title compounds occurs, identifying their volatile decomposition products. Understanding of the detailed thermal behaviour of disubstituted annelated triazinones with analgesic and anticancer activities will be an essential part of their further characterisation and, at the same time, an important contribution to the current state of knowledge. The thermal characterisation of these pharmacologically important compounds with prospective (medical and analytical) use will be very important for the drug development process and possible further pharmaceutical application. Additionally, the results of the present paper will be helpful in a further evaluation of the most promising compounds as possible drugs. In our studies we applied thermogravimetry-differential scanning calorimetry (TG-DSC) and thermogravimetry coupled with Fourier transform infrared (TG-FTIR) spectroscopy, since these combined and coupled thermal analysis techniques not only enable the reliable interpretation of any phenomenon occurring with mass and energy changes in the heated samples of the investigated molecules but also allow for the reliable controlling their purity (monitoring the content or lack of residual solvents used in the synthesis process) [6]. Our studies will be of practical usefulness in the case of the pharmaceutical approval of this class of annelated triazinones. Based on the results it will be possible to establish the optimal storage and processing conditions for all molecules in this important class. Furthermore, in the event of need for thermal utilisation of these compounds after their shelf life, based on the obtained results, it will be possible to determine such thermal conditions for their controlled combustion process that the released volatile decomposition products do not pollute the environment.

## 2. Results and Discussion

Differential scanning calorimetry (DSC) and thermogravimetric analysis (TG) are widely used analytical methods whose employment in the study of pharmaceuticals and potential drugs is constantly increasing. DSC and TG rely on a linear heating/cooling of the tested samples and measurement of the temperatures and heats of phase transitions or mass losses occurring under these conditions. These methods make it possible to study physical processes, including melting, sublimation, dehydration, crystallisation, polymorphic transformations, and glass transition, as well as chemical processes such as thermal degradation. The combination of thermogravimetry (TG) with Fourier transform infrared spectroscopy (FTIR) gives a perfect tool (TG-FTIR) for the detection and identification of volatile products evolved during the heating of samples. The TG-FTIR technique belongs to the favoured evolved gas analysis methods used in thermal analysis [17]. The simultaneous observation of mass changes along with recorded decomposition products of compounds in nitrogen allows not only the examination of their thermal stability in an inert atmosphere but also proposition of the mechanism of their decomposition.

### 2.1. Thermal Characterisation of Annelated Triazinones in Air (**1–9**)

**Figure 2:**
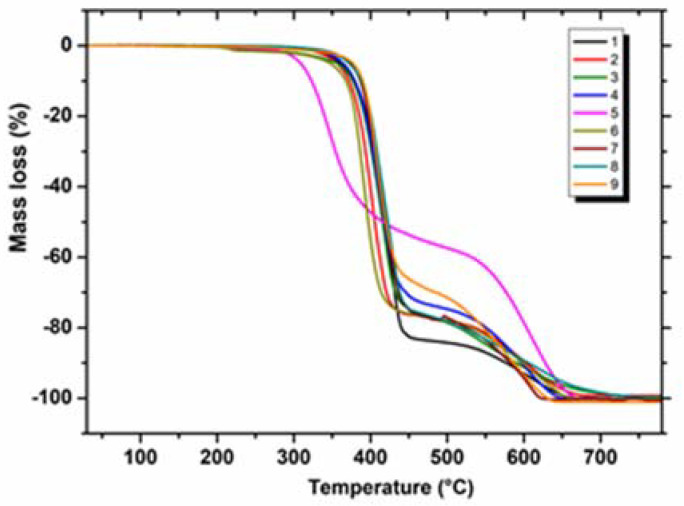
TG curves for compounds **1–9** (an air atmosphere).

**Figure 3:**
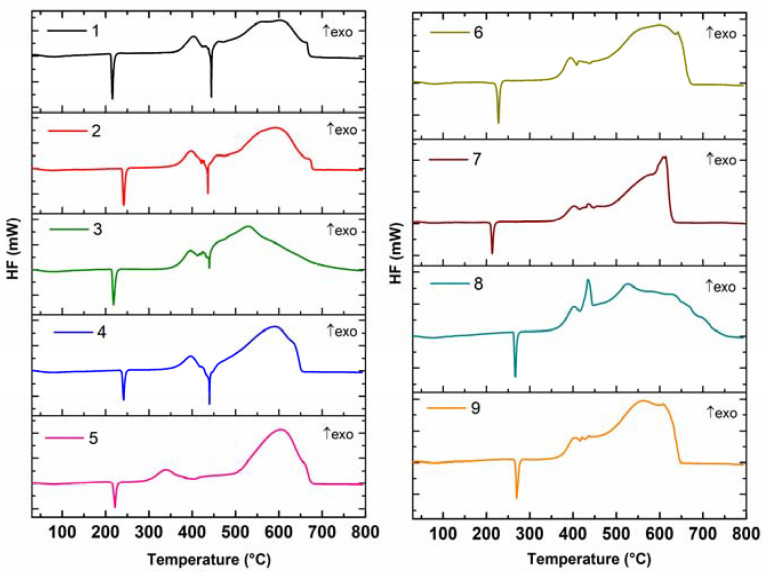
DSC curves for compounds **1–9** (in an air atmosphere).

All the samples of annelated triazinones were characterised by TG mass loss and DSC curves (Figure 2 and Figure 3). The DSC method was used to confirm the purity of all the crystalline substances that were investigated (**1–9**). The solid-liquid phase transition (melting) for all the tested compounds (**1–9**) was described by one sharp endothermic peak on their DSC curves, which proved their absolute purity. This is as expected because the previous spectroscopic, crystallographic, and chromatographic investigations [1,2,3,4] confirmed that all the compounds are homogeneous, crystalline substances of high purity. The advantage of all the molecules subjected to thermal studies is that they were crystalline substances with sharp melting points. In light of thermal analysis reports, the crystalline pharmaceutical substances are generally more stable than amorphous drugs, and therefore upon storage they do not have a tendency to undergo polymorphic transformations with possible loss of their bioactivity [6].

Absolute purity is an advantageous thermal property of potential medicinal substances. The parent structure **1**—which contains no substituent attached to the phenyl moiety—has a melting point (T_peak_) of 216 °C. Replacing a hydrogen atom at the benzene ring with a methyl group, or a methoxy substituent, or one chlorine atom (in the *ortho* or *para* position), or two chlorine atoms increases the melting point. Exceptionally, the substitution in the *meta* position at the phenyl ring, which is likely to make the molecule less symmetrical, results in a decrease in the melting point of compound **7** compared to the parent structure **1**. Compounds **8** and **9** have the highest melting points. In the case of **8** (the *para*-Cl derivative) and **9** (the *meta*,*para*-Cl_2_ derivative) an increase in the melting point is significant (from 50 to even 54 °C) compared to the parent structure **1** (Table 1). For molecules such as compound **5** (the *ortho*-OCH_3_ derivative) and **6** (the *ortho*-Cl derivative), the melting points are 221 and 227 °C, respectively. Compounds **2** and **4**, which have a methyl group in the *ortho* or *para* position, respectively, melt at the same temperature (T_peak_) of 241 °C. In turn, the *meta*-methyl derivative (compound **3**) melts at a temperature two degrees higher than the parent structure **1**. Compound **7** is one of six molecules with a lower melting point than the parent structure without a substituent at the phenyl ring (Table 1). The observed differences between the melting points of particular derivatives in the investigated class of annelated triazinones may be explained in light of the present knowledge. According to the rule known for benzene derivatives, in general, the melting points of particular benzene derivatives do enhance with increasing molecular mass. An exception is the case where an increase in the molar mass of a certain benzene derivative is evoked by a substitution which makes the molecule less symmetrical [18,19]. 

Melting enthalpy values (ΔH_m_) obtained in oxidative conditions range from 26.77 kJ mol^−1^ to 39.10 kJ mol^−1^ (Table 1). The highest value of ΔH_m_ was observed for compound **9** containing the 3,4-Cl_2_ substitution at the benzene ring. At the same time, this compound had the highest melting point among all the annelated triazinone derivatives with a disubstituted bicyclic scaffold. Additionally, this molecule is one of the two compounds that are the most thermally stable in an air atmosphere. Interestingly, the lowest melting enthalpy among all the synthesized compounds is shown by the derivative bearing the chlorine atom in the *ortho* position at the phenyl ring (compound **6**). Hence, it is seen that the least heat energy (26.77 kJ mol^−1^) is required to convert a mole of a solid at its melting point into a liquid without an enhancement in temperature for this compound.

After the melt transition, the baseline returns to the same position as the pre-melt baseline (Figure 3). The baseline after melting changes its slope as samples (**1–9**) begin to decompose in the temperature range from 241 to 296 °C. Two main stages of decomposition can be distinguished on the TG curves (Figure 2). The first mass loss of 53.95–83.56% for all the compounds (**1–9**) was associated with the formation of unstable decomposition products. For the first four compounds (**1–4**) at this stage, the DSC curve shows both the endo-degradation processes with clearly defined peaks (T_p1_ 444, T_p2_ 437, T_p3_ 440, T_p4_ 441 °C) and the exo-degradation processes. In turn, for the remaining compounds (**5–9**) the peaks resulting from the exo-degradation processes are visible on their DSC curves (Figure 3).

The last stage of degradation occurs above 453 °C for compound **5**, which begins to decompose at the lowest temperature for this stage, while compound **7** (which has the highest temperature for this stage) decomposes above 491 °C. The final decomposition temperature, at which a plateau occurred on the TG curve, was in the range of 632–768 °C (Table 1). The above data show that compound **3** is the most thermally stable because its molecule degrades at the highest temperature.

Concluding, all the tested compounds revealed high thermal stability in air. Each analysed sample of pure substance revealed one sharp DSC peak characterising its solid–melting phase transition. Thus, the DSC results presented in this paper clearly indicate the phase purity of these compounds, as evidenced by the clear endothermic effect associated with their melting. The melting points were dependent on the structure of the analysed molecules. Furthermore, each compound did not undergo any polymorphic transformations. It can therefore be supposed that their storage in a temperature range of 20–45 °C will not result in losing of their shelf life [15,16].

### 2.2. Thermal Characterisation of Annelated Triazinones in Nitrogen (**1–9**)

**Figure 4:**
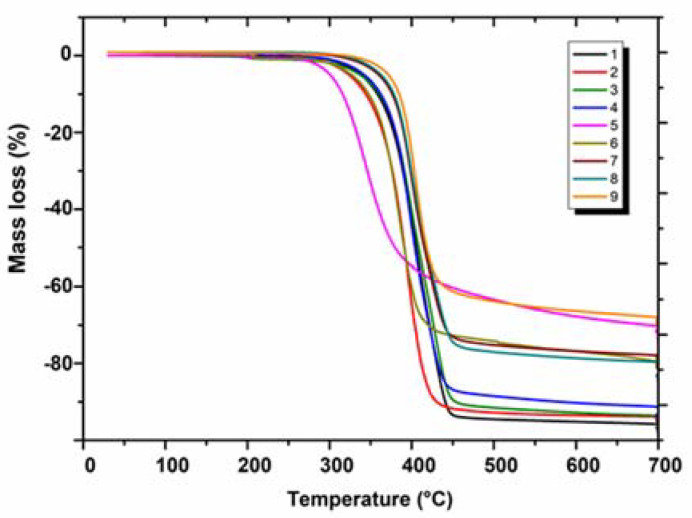
TG curves of **1–9** compounds (in a nitrogen atmosphere).

The thermal stability of the investigated compounds (**1–9**) in a nitrogen atmosphere changes in the following order: **6** (201 °C) < **5** (207 °C) < **2** (266 °C) ≈ **7** (268 °C) < **1** (279 °C) < **4** (285 °C) ≈ **3** (286 °C) < **9** (294 °C) < **8** (301 °C) (Figure 4). The lowest thermal stability in both atmospheres is exhibited by compounds **6** and **5** which contain in their structures the chloro or methoxy group in the *ortho* position at the phenyl moiety, respectively. The presence of the methyl group in position *ortho* (**2**) and the chlorine atom in position *meta* (**7**) significantly increases the thermal stability of compounds. The higher thermal resistance, in comparison to the above-mentioned compounds, exhibits the parent structure **1,** which is unsubstituted at the phenyl moiety. Introducing the methyl group in position *meta* or *para* significantly enhances the stability of compounds **3** and **4**. The highest thermal stability is shown by two molecules: one with chlorine substituent in position *para* (compound **8**) and another with chlorine atoms in positions *ortho* and *para* (compound **9**). A similar impact of the substituents attached at the phenyl moiety on the thermal stability was observed in a class of 3-(thiophen-2-yl)-8-(R-phenyl)-7,8-dihydroimidazo[2,1-*c*][1,2,4]triazin-4(6*H*)-ones [13]. In turn, quite a different influence of chlorine substituents on the thermal stability was observed in previously reported ethyl 2-(4-oxo-8-substituted-4,6,7,8-tetrahydroimidazo[2,1-*c*][1,2,4]triazin-3-yl)acetates [20]. 

The tendency of changes in the thermal stability of the tested compounds in nitrogen (depending on the type and position of substituents in the phenyl ring) was almost the same as in air, taking into account the temperature where the tested samples start to decompose (Table 1).

As can be seen from the analysis of TG curves (Figure 4), the thermal degradation of compounds **1–9** occurs in one step in the relatively narrow temperature range of 202–490 °C where the majority of sample masses are lost. Above 490 °C only slight mass changes of 2–7% take place, up to 700 °C. The total degradation of compounds in nitrogen occurred in different grades as was reflected in the masses of solid residues (unburnt carbon) which changed as follows: **1** (3.99%) < **3** (5.66%) < **2** (5.99%) < **4** (8.74%) < **8** (11.70%) < **6** (12.36%) < **7** (13.46%) < **9** (25.00%) < **5** (29.8%).

**Figure 5:**
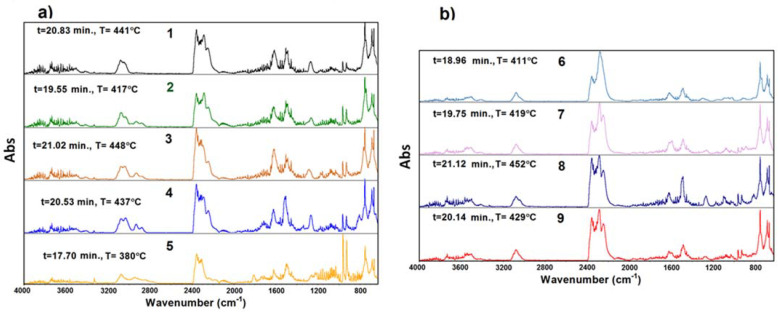
The FTIR spectra of volatile decomposition products for compounds **1-5** (**a**) and **6-9** (**b**) recorded in nitrogen at a temperature of the highest emission of gases.

Pyrolytic decomposition of the investigated annelated triazinones in nitrogen was accompanied by the evolution of volatile substances being the products of chemical bond disruption. Carbon dioxide and water molecules of different intensities were released from the beginning and up to the end of the compounds’ degradation. The multipeak bands in the wavenumber ranges of 4000–3200 and 1800–1300 cm^−1^ were assigned to the stretching and deformation vibrations of water molecules. The carbon dioxide gives several bands in the range of 2400–2250 cm^−1^ and a single sharp band at 668 cm^−1^ due to its stretching and deformation vibrations [21]. At a slightly higher temperature, further chemical compounds are liberated. As can be deduced from the TG curves of the examined compounds, the H_2_O and CO_2_ appeared earliest in the case of **5,** which coincides well with the low thermal stability of this compound. For the remaining compounds, these volatile molecules are recorded above 14 min. The highest intensity of gas release is observed in the temperature range of 380–450 °C. The single FTIR spectra recorded at the temperature of the highest emission of gases are summarized in Figure 5. It is worth noting that these temperatures are in good agreement with the stability order of compounds **1–9** given above (i.e., the most intense evolution appears at the highest temperature for the compound of the highest stability).

The detailed analysis of infrared spectra allows us to draw some conclusions about the decomposition mechanism of the investigated compounds. Particularly interesting is the analysis of infrared spectra within the range of 2400–2200 cm^−1^. Except bands from water and carbon dioxide molecules, all the recorded FTIR spectra also exhibit bands of different intensity at 2286, 2279, and 2251 cm^−1^, which was ascribed to the stretching vibrations of NCO groups most probably due to the evolution of HNCO and its derivatives such as phenyl isocyanate. An additional confirmation of this statement is the presence of diagnostic bands at higher and lower wavenumbers. In the ranges of 3650–3480 cm^−1^ and 850–650 cm^−1^, relatively weak bands appear assigned to the stretching vibrations of H-N groups and the bending vibrations of HNCO and NCO groups from isocyanic acid [22]. At the same time, quite strong bands observed at 1616, 1507, 754, 746, and 687 cm^−1^ can be assigned to the stretching of C_Ar_C_Ar_, out-of-plane C-H, and out-of-plane ring bending vibrations, respectively. These bands are indicative of the liberation of benzene derivatives. Moreover, all the compounds exhibit a broad band in the range of 3150–3000 cm^−1^ which results from the stretching vibrations of C_Ar_H groups from aromatic moieties. The FTIR spectra of compounds **2–5** display broader bands in the range of 3100–2800 cm^−1^ with submaxima at 3076, 3033, 2936, and 2920 cm^−1^ derived from the asymmetric and symmetric stretching vibrations of the methyl group. These bands confirm that the decomposition process of the studied compounds leads to the evolution of phenyl substituents in form of different species.

**Figure 6:**
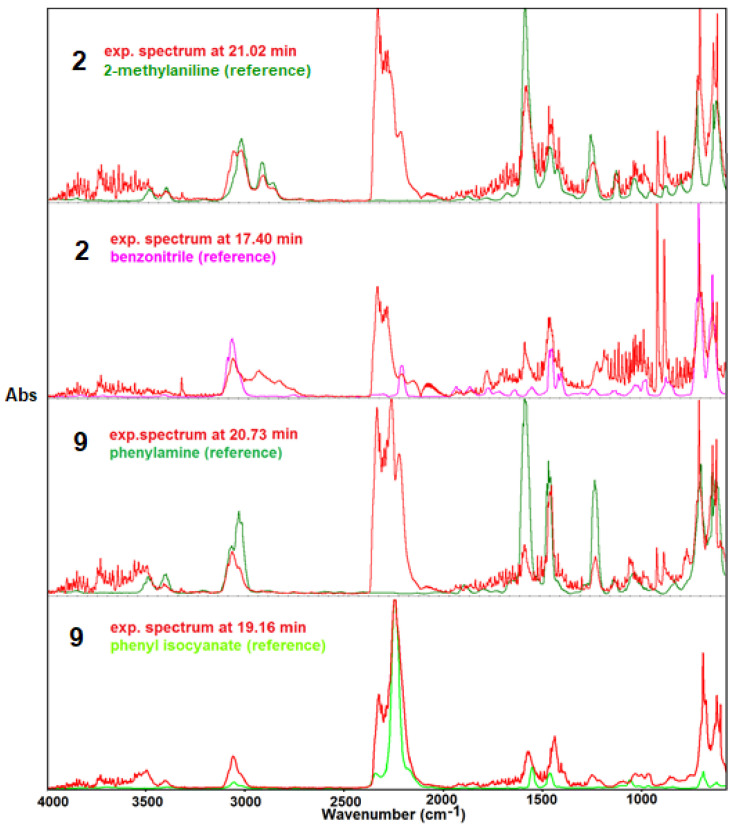
Experimental spectra of compounds **2** and **9** along with reference spectra [23,24,25,26].

Almost all spectra also show characteristic doublet bands at 956 and 930 cm^−1^ derived from the evolved NH_3_ and/or N_2_H_4_. Aside from the attendance of the above identified molecules/groups, it worth taking into consideration the ratio of the stretching vibrations intensities of CO_2_/NCO (2400–2200 cm^−1^). Vibrations from CO_2_ dominate in the FTIR spectra of compounds **1–5**, whereas vibrations from molecules containing NCO moieties dominate in the FTIR spectra of compounds **6–9**. The FTIR spectra of **1–5** also display very weak vibrations from carbon monoxide in the range of 2200–2100 cm^−1^. Taking into consideration the above detected and identified molecules/groups, it can be said that the mechanisms of the decomposition process in **1–5** and **6–9** molecules occur in different ways. The parent compound **1** and those containing a methyl or methoxy group in the structure (**2–5**) degrade up to 450 °C, with the release most likely of volatile products such as methylaniline (isomers, for methyl derivatives), *o*-anisidine, aniline, benzene, and/or benzenecarbonitrile [23,24,25,26]. The gaseous mixtures of the released molecules from compounds **6–9** do not contain toluidine but in fact the remaining hydrocarbons and greater amounts of HNCO and phenyl isocyanate. The suggested composition of gaseous mixtures was made based on the comparison between the experimental and reference spectra of the identified compounds (Figure 6). The presence of CO_2_, H_2_O, NH_3_/N_2_H_4_, HNCO, and its derivatives in the volatile decomposition products confirmed not only that the cleavage of bonds between substituents and the triazine moiety took place but also that those inside the triazine template were broken. 

At higher temperature (above 450 °C), the FTIR spectra of gas phase products are dominated mainly by bands derived from carbon oxides and water molecules evolved from solid residues. The intensities of carbon monoxide significantly increased, especially for compounds **6–9** for which the total mass losses were lower in comparison to the remaining compounds. The thermal degradation of compounds **6**, **8**, and **9** leads also to the evolution of HCl which gives multipeak bands within the range of 3100–2600 cm^−1^.

### 2.3. Evaluation of the Effect of the Tested Compounds (**1–9**) on Erythrocytes

The tested compounds are potential analgesic and anticancer agents [1,2], which are characterised by high thermal stability (reported in this paper) and optimal lipophilicity values well-correlated with their biopharmaceutical properties (relevant to pharmacokinetics) [3,4]. Hence, it is reasonable to determine their safety/toxicity profile. Erythrocytes are a good model to study the cytotoxicity of pharmacologically important compounds in the preclinical phase of drug development. For this purpose, ex vivo studies were performed to assess the effect of disubstituted annelated triazinones (**1–9**) on the important and, simultaneously, most numerous blood cells, i.e., red blood cells. 

By examining the effect of all the compounds on red blood cells, their good haemocompatibility was confirmed. It has been proved that all the investigated disubstituted annelated triazinones (**1–9**) are safe for erythrocytes, as none of them caused significant haemolytic effects (the haemolytic activity less than 5% compared to the positive control, i.e., Triton X-100 causing complete haemolysis) (Table 2). The lack of haemolytic properties is particularly important due to the potential therapeutic usefulness of these drug candidates.

In turn, by exposing erythrocytes (pre-incubated with disubstituted annelated triazinones) to reactive oxygen species causing their damage, the ability of the compounds to inhibit oxidative haemolysis was assessed. The antihaemolytic properties of the studied molecules were tested on red blood cells exposed to peroxyl radicals generated by AAPH (2,2′-azobis(2-methylpropionamidine) dihydrochloride) or hydrogen peroxides, and compared with the activity of standard antioxidants, i.e., ascorbic acid or Trolox. In the class of annelated triazinones, compounds **3**, **6**, and **9** showed the best protection of red blood cells against oxidative damage (Table 2). The strongest inhibition of peroxyl radical-induced haemolysis was observed after incubation of erythrocytes with derivatives substituted on the phenyl ring with a methyl group in the *meta* position (compound **3**) and a chlorine atom in the *ortho* position (compound **6**). Their antihaemolytic activity was 81 and 80%, respectively, of that of ascorbic acid. In turn, the most active in preventing erythrocyte damage caused by hydrogen peroxides proved to be a derivative with two chlorine atoms in the *meta* and *para* positions of the phenyl moiety (compound **9**), whose activity was 83% of that of Trolox. The efficient protection of red blood cells from oxidative damage, such as in the case of compounds **3**, **6**, and **9**, is a beneficial property of drug candidates.

### 2.4. Assessment of the Risk of Side Effects of All the Compounds (**1–9**)

The risk of serious adverse effects may be a major problem in the case of new potential medicines. Therefore, this risk has to be already assessed in the preclinical phase of drug development. Mutagenic, tumorigenic, irritating, and reproductive effective molecules cannot be tested on animals. In order to predict the possible appearance of adverse side effects in our class of disubstituted annelated triazinones, we have decided to apply a useful in silico tool–the OSIRIS Property Explorer (available online at http://www.organic-chemistry.org/prog/peo/, accessed on 13 May 2023). The risk predictor is able to locate each structural fragment which gives rise to toxicity alerts, if it is present in the molecule studied. A collection of these fragments was taken from the Registry of Toxic Effects of Chemical Substances database, containing above 16,280 of chemical substances that reveal mutagenic, tumorigenic, irritant, and reproductive effects, and more than 3340 commercially approved molecular pharmaceutics as a control group. The result of predicting the risk of an adverse effect is colour-coded as follows: green colour—no risk (score 1.0); yellow colour—medium risk (score 0.8), and red colour—high risk (score 0.6). Our annelated triazinones (**1–9**) have been found in silico to be non-mutagenic, non-carcinogenic, non-irritating, and non-reproductive effective (Table 3). This may most likely be because there are no structural fragments in their molecules that cause toxicity alerts.

### 2.5. Predicting Molecular Targets for the Investigated Compounds (**1–9**)

The possible molecular targets for disubstituted annelated triazinones (**1–9**) were assessed using the Molinspiration Cheminformatics free web server (available online at www.molinspiration.com, accessed on 13 May 2023). The investigated small molecules (**1–9**) were virtually screened as potential G-protein-coupled receptor (GPCR) ligands, kinase inhibitors, enzyme inhibitors, ion channel modulators, nuclear receptor ligands, and protease inhibitors (Table 4). 

Considering the bioactivity scores shown in Table 4, all annelated triazinones have the highest probability of being active as GPCR-modulating ligands, kinase inhibitors, and enzyme inhibitors, although they can act on a variety of molecular targets. The structural similarity of the studied compounds to biogenic nucleic bases suggests their ability to inhibit enzymes participating in metabolic pathways [27]. As false building blocks, these molecules may be capable of replacing biogenic nucleobases during the DNA synthesis phase, leading to a lethal synthesis. Additionally, the previous experimental findings proved that structurally related compounds can exhibit antagonistic activity towards two subtypes of adenosine receptors (such as A_2A_ AR and A_2B_ AR) belonging to GPCRs [28].

**Table 4:** The bioactivity score of all the investigated compounds (**1–9**) for various molecular targets.

Compound	Bioactivity Score					
	GPCR Ligand	Kinase Inhibitor	Enzyme Inhibitor	Ion Channel Modulator	Nuclear Receptor Ligand	Protease Inhibitor
**1**	−0.16	−0.34	−0.35	−0.62	−0.80	−0.82
**2**	−0.20	−0.31	−0.37	−0.67	−0.79	−0.85
**3**	−0.19	−0.37	−0.42	−0.69	−0.78	−0.83
**4**	−0.18	−0.37	−0.40	−0.68	−0.78	−0.82
**5**	−0.20	−0.31	−0.35	−0.75	−0.76	−0.86
**6**	−0.07	−0.20	−0.39	−0.61	−0.81	−0.82
**7**	−0.15	−0.33	−0.40	−0.61	−0.78	−0.84
**8**	−0.14	−0.34	−0.37	−0.60	−0.77	−0.81
**9**	−0.13	−0.31	−0.38	−0.58	−0.73	−0.79

GPCR—G-protein-coupled receptor. Interpretation of the bioactivity score: >0.00: active; −5.00–0.00: moderately active; <−5.00: inactive [29,30,31].

## 3. Materials and Methods

### 3.1. A Set of the Investigated Disubstituted Annelated Triazinones (**1–9**)

Disubstituted annelated triazinones, i.e., 3-phenyl-8-(R-phenyl)-7,8-dihydroimidazo[2,1-*c*][1,2,4]triazin-4(6*H*)-ones (**1–9**) were available from our synthetic studies that had been patented and published previously [1,2]. Briefly, they were synthesised in the two general synthetic approaches reported previously by reacting 2-hydrazinylidene-1-(R-phenyl)imidazolidine with oxo(phenyl)acetic acid or ethyl oxo(phenyl)acetate. The structures of all the molecules recruited (**1–9**) for the purposes of their thermal investigations have been confirmed by their consistent spectroscopic data [1,2]. All the compounds have been synthesised as solid substances possessing sharp melting points and microanalyses within ±0.4 of the calculated values [1,2]. The purity and homogeneity of all the samples intended to thermal studies were confirmed in previous chromatographic investigations [3,4]. 

### 3.2. Thermal Analysis Methods

The thermogravimetric (TG) and differential scanning calorimetry (DSC) measurements were carried out using the SETSYS 16/18 analyser (Setaram, Caluire, France). The samples of masses 6–9 mg were heated in the alumina crucibles from room temperature up to 1000 °C at a heating rate of 10 °C min^−1^ in the flowing air atmosphere (12.5 cm^3^ min^−1^). The TG curves in nitrogen were recorded using the Q5000 thermal analyser (TA Instruments, New Castle, DE, USA) coupled with the Nicolet 6700 spectrophotometer (Thermo Scientific, Waltham, MA, USA) for infrared spectra recording. Samples of 25–30 mg were heated in the dynamic nitrogen atmosphere (25 cm^3^ min^−1^) at a heating rate of 10 °C min^−1^. The samples were heated from room temperature up to 700 °C in open platinum crucibles. The transfer line was heated up to 250 °C while the gas cell of spectrophotometer was heated up to 240 °C. 

### 3.3. Investigation of the Effect of Compounds **1–9** on Erythrocytes

The effect of disubstituted annelated triazinones on erythrocytes was tested in the haemolytic assay as well as in the oxidative haemolysis inhibition assay, according to the procedures described earlier [20]. Blood used for the tests was collected from rats (male Wistar rats; 8 weeks old; 240–250 g) kept at the Experimental Medicine Centre of the Medical University of Lublin, Poland. 

## 4. Conclusions

The samples of annelated triazinones (**1–9**) revealed high thermal stability in oxidative and inert conditions and were characterised by high purity (as evidenced by the clear endothermic effect associated with their melting). In addition, they did not undergo any polymorphic transformations. Hence, these favourable thermal features make them suitable for pharmaceutical applications, as they would not require any special conditions during their storage in a wide range of temperatures (20–45 °C). The presented results are of importance in the detailed thermal characterisation of this promising class of pharmacologically relevant polynitrogenated heterocycles. They allow us to evaluate the thermal properties and events (characterised by the enthalpy change and by the temperature range) of molecules in a preclinical phase of drug development. The results shown are also important from a practical point of view. They will allow us to determine the optimal conditions for the storage and processing of these potential pharmaceutics. In addition, they will enable the selection of appropriate temperatures and filters during their controlled thermal utilisation, so that the gaseous products of their decomposition do not pollute the environment. It should be highlighted that all the compounds (**1–9**) proved to be safe for red blood cells. In turn, molecules **3**, **6**, and **9** protected erythrocytes from oxidative damage, and therefore these structures should be helpful in the development of preventive agents against free radical-mediated diseases.

## Figures and Tables

**Table 1:** Thermal data for compounds **1–9** (an air atmosphere).

Sample	Melting Process			Decomposition Process		
	T_onset_ [°C]	T_peak_ [°C]	ΔH_m_[kJ·mol^−1^]	Step 1		Step 2
				ΔT_1_ [°C]	Δm_1_ [%]	ΔT_2_ [°C]
**1**	212	216	29.18	288–471	83.56	471–670
**2**	238	241	34.12	279–454	76.44	454–676
**3**	215	218	32.58	253–460	75.56	460–768
**4**	237	241	31.04	283–473	73.31	473–670
**5**	216	221	36.99	241–453	53.95	453–685
**6**	223	227	26.77	247–464	76.69	464–668
**7**	210	213	31.42	284–491	78.00	491–632
**8**	262	266	28.85	296–460	75.55	460–745
**9**	267	270	39.10	290–455	67.00	455–650

T_onset_—onset temperature of endothermic effect; T_peak_—melting peak temperature; ΔH_m_—melting enthalpy; ΔT_1_, ΔT_2_—temperature ranges of decomposition stages; Δm_1_—mass loss.

**Table 2:** Haemolytic and antihaemolytic activities of the investigated compounds (**1–9**).

Compound/Control	Haemolytic Activity (%) ^A^	Inhibition (%) of Oxidative Haemolysis	
		Induced by AAPH ^B^	Induced by H_2_O_2_ ^C^
**1**	2.47 ± 0.12	55 ± 4.4	59 ± 6.1
**2**	4.04 ± 0.13	42 ± 4.5	45 ± 4.0
**3**	2.14 ± 0.17	81 ± 9.3	77 ± 7.0
**4**	3.79 ± 0.15	60 ± 5.7	58 ± 4.4
**5**	4.12 ± 0.23	48 ± 3.2	34 ± 5.1
**6**	2.39 ± 0.11	80 ± 6.6	76 ± 8.8
**7**	4.78 ± 0.14	39 ± 4.5	60 ± 5.5
**8**	3.46 ± 0.28	69 ± 7.0	57 ± 4.2
**9**	2.39 ± 0.17	74 ± 5.4	83 ± 7.1
Triton X-100	100	-	-
Ascorbic acid	-	100	-
Trolox	-	-	100

AAPH—2,2′-azobis(2-methylpropionamidine) dihydrochloride; H_2_O_2_—hydrogen peroxide; Trolox—6-hydroxy-2,5,7,8-tetramethylchroman-2-carboxylic acid; ^A^—haemolytic activity of compounds **1–9** in relation to a positive control, i.e., 1% Triton X-100 solution; ^B^—antihaemolytic activity (in the model of rat erythrocytes exposed to AAPH) of compounds **1–9** in relation to ascorbic acid; ^C^—antihaemolytic activity (in the model of rat erythrocytes exposed to H_2_O_2_) of compounds **1–9** in relation to Trolox. Compounds were tested at a concentration of 0.15 mM. Data (from three independent experiments) are shown as the mean ± standard deviation.

**Table 3:** Risk assessment of adverse side effects by OSIRIS Property Explorer for the studied compounds (**1–9**).

Compound	Mutagenicity	Tumorigenicity	Irritating Effects	Reproductive Effects
**1**	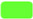	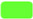	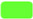	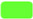
**2**	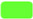	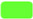	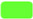	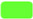
**3**	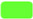	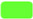	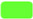	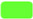
**4**	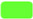	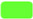	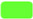	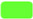
**5**	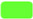	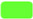	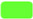	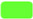
**6**	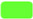	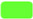	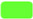	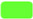
**7**	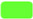	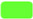	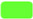	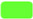
**8 ^a^**	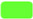	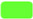	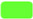	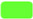
**9**	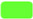	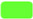	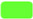	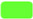

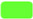
—no risk, score: 1.0; ^a^—data published in Ref. [5].

## Data Availability

The data presented in this study are available on request from the authors.

## Sample Availability

Samples of the compounds **1**–**9** are available from the authors.
